# Dual-Branch Graph Learning with Frequency Gating for Industrial Sensor Anomaly and Cyberattack Detection

**DOI:** 10.3390/s26113607

**Published:** 2026-06-05

**Authors:** Tong Zhao, Wei Yang, Yu Yao

**Affiliations:** 1 School of Computer Science and Engineering, Northeastern University, Shenyang 110819, China; zhaot8@mails.neu.edu.cn; 2 College of Software, Northeastern University, Shenyang 110169, China; yangwei@mail.neu.edu.cn

**Keywords:** industrial sensor, anomaly detection, cyberattack detection, graph learning, time series, frequency gating, Gaussian kernel

## Abstract

Industrial sensor systems are increasingly vulnerable to both physical anomalies and cyberattacks, while their collected time series typically present complex periodic and non-stationary characteristics, along with dynamic spatial dependencies among sensors. To address these issues, this paper proposes a dual-branch graph learning framework with frequency gating for simultaneous industrial sensor anomaly and cyberattack detection. The model first divides the input time series into multiple patches and decomposes each patch into periodic and non-stationary components via frequency analysis. Two graph isomorphism network branches, namely periodic GIN (P-GIN) and non-stationary GIN (NS-GIN), are designed to model the spatial dependencies of the two components separately, where the graph structure is adaptively learned using a Gaussian kernel-based mechanism. Furthermore, a frequency gating module is introduced in the non-stationary branch to enhance the representation of abnormal and attack-related features. Hierarchical temporal encoding is performed via intra-patch attention and inter-patch attention to capture both local and long-range temporal dependencies. Extensive experimental results on real-world industrial sensor datasets demonstrate that the proposed method achieves superior performance compared with state-of-the-art methods in both anomaly detection and cyberattack detection tasks.

## 1. Introduction

Cyber–physical systems (CPSs) have become an indispensable foundation of modern society, with widespread deployments across critical industrial sectors [1]. As core components of CPSs, industrial control systems (ICSs) and the Industrial Internet of Things (IIoT) play a pivotal role in monitoring and controlling critical infrastructure and industrial processes, seamlessly integrating physical operations with computational intelligence, networked sensors, actuators, and controllers [2]. While these technologies significantly enhance operational efficiency, automation levels, and real-time decision-making, their open and interconnected nature also exposes industrial sensor networks to unprecedented cybersecurity risks and physical anomalies. Industrial sensors, as the “perceptual terminals” of CPSs, continuously collect and transmit real-time operational data that directly reflects system states; however, cyberattacks (e.g., data tampering and denial-of-service attacks) and physical anomalies (e.g., equipment wear and component failure) often introduce irregular high-frequency variations into the time series, which are difficult to capture using conventional detection methods [3].

From a structural perspective, industrial sensor systems are inherently graph-structured: sensors are physically interconnected through pipelines, control loops, and communication networks, forming complex spatial dependency patterns [4]. Such dependencies are often dynamic and nonlinear. For example, a pump failure may propagate abnormal fluctuations to distant tanks and valves through hydraulic coupling [5]. Effectively modeling these spatial relations is therefore critical for accurate anomaly and cyberattack detection.

Graph neural networks (GNNs) have recently emerged as a powerful paradigm for modeling relational data, offering unique advantages in capturing both localized and system-wide dependencies [6]. Unlike conventional temporal models that treat sensors as independent variables, GNNs can explicitly encode topological constraints and physical interactions, making them particularly suitable for industrial environments where anomalies often manifest as structural deviations rather than isolated pointwise outliers. Moreover, adaptive graph learning enables the model to infer latent sensor correlations directly from data, avoiding reliance on manually defined topologies that may be incomplete or outdated.

Existing anomaly detection methods for industrial sensor time series still face several key limitations [7,8]. First, most methods fail to effectively disentangle periodic (normal operational patterns) and non-stationary (abnormal/attack-related) components in the time series, making it hard to distinguish subtle high-frequency abnormal signals from normal periodic fluctuations [9]. Second, traditional graph-based methods rely on predefined fixed graphs to model sensor spatial dependencies, which cannot adapt to the dynamic correlations between sensors in complex industrial scenarios [10,11]. Third, few methods integrate effective temporal modeling mechanisms to capture long-range dependencies across time steps, further limiting their detection accuracy [12]. To address these challenges, there is an urgent need to develop a unified detection framework that can leverage frequency-domain characteristics, dynamic spatial modeling, and temporal sequence learning for enhanced industrial sensor anomaly and cyberattack detection.

To fill this research gap, this paper proposes a dual-branch graph learning framework with frequency gating for industrial sensor anomaly and cyberattack detection. We posit that non-stationarity constitutes an inherent characteristic of industrial signals; consequently, our focus is on isolating anomalous deviations within non-periodic trends rather than treating non-stationarity as a proxy for anomalies. The framework achieves this by explicitly decomposing the series into periodic and non-stationary branches, further augmented by dynamic spatial and temporal modeling. Specifically, the input industrial sensor time series is first segmented into non-overlapping patches to balance local feature extraction and computational efficiency. Each patch is then decomposed into periodic and non-stationary components via frequency-domain analysis: the periodic component reflects stable system operation cycles, while the non-stationary component contains high-frequency anomalies and attack-related fluctuations. Two dedicated graph isomorphism network (GIN) [13] branches (periodic GIN and non-stationary GIN) are designed to model the spatial dependencies of these two components separately, where a Gaussian kernel-based graph structure learning (GSL) [14] module adaptively learns dynamic sensor correlations without relying on predefined graphs, realizing the dynamic graph characteristic of the proposed method. A frequency gating (FreqGate) module is integrated into the non-stationary GIN branch to suppress irrelevant low-frequency noise and amplify critical high-frequency abnormal features. Additionally, gated recurrent unit (GRU) [15] modules are incorporated into both branches to model temporal evolution within patches and long-range dependencies across patches, complemented by hierarchical intra-patch and inter-patch attention to enhance temporal encoding.

The main contributions of this paper can be summarized as follows:

1. A dual-branch graph learning framework is proposed to explicitly disentangle periodic and non-stationary components, enabling targeted modeling of normal and abnormal patterns in industrial sensor time series;
2. A Gaussian kernel-based GSL module is introduced to realize dynamic graph learning, adaptively capturing time-varying spatial dependencies between sensors and improving the model’s generalization ability;
3. The integration of FreqGate and GRU enhances the model’s capability to capture high-frequency abnormal features and long-range temporal dependencies, addressing the limitations of existing methods;
4. Extensive experiments on real-world industrial datasets demonstrate that the proposed method outperforms state-of-the-art approaches in both anomaly and cyberattack detection tasks, providing a reliable solution for industrial sensor network security.

## 2. Related Works

### 2.1. Multivariate Time-Series Anomaly Detection

Time-series anomaly detection primarily employs prediction-based or reconstruction-based methods. Early prediction approaches leveraged LSTM [16] and transformer [17] architectures for sequential modeling. Subsequent transformer enhancements include Autoformer [18], Crossformer [19], and FEDformer [20], which integrate seasonal decomposition to extract periodic and trend components. For parameter efficiency, convolutional networks emerged (e.g., TimesNet [21] and PatchTST [22]), capturing local temporal features. Recent research rediscovered MLP’s (multilayer perceptron) efficacy, yielding architectures like DLinear [23]. With the emergence of deep learning-based time-series analysis methods integrated with frequency-domain techniques, KfreqGAN [24] trains an adversarial forecasting model using auxiliary information derived from the frequency domain for anomaly detection.

While reconstruction-based methodologies demonstrate broader applicability than prediction-based approaches in industrial anomaly detection, significant technical evolution has occurred across several paradigms. DAGMM [25] pioneered the integration of autoencoders with Gaussian mixture models, enhancing density estimation capabilities. Subsequent approaches addressed data sparsity through variational frameworks like LSTM–VAE [26] and OmniAnomaly [27]. Concurrently, generative adversarial networks emerged as dominant solutions for handling data imbalance, with MAD-GAN [28] and USAD [29] leveraging adversarial learning for robust reconstruction. More recent innovations include contrastive learning architectures such as DCdetector [30], which constructs discriminative embedding spaces isolating anomalies, and frequency-domain techniques exemplified by CATCH [31] that learn cross-dimensional dependencies through spectral analysis. This progression highlights continual refinement in addressing core challenges of data scarcity, feature representation, and multivariate dependency modeling.

In addition to prediction-based and reconstruction-based anomaly detection methods (e.g., LSTM and GANs), classification-based and probability-based approaches have garnered widespread attention in the research community, primarily due to their relatively simple model structure and ease of implementation. For instance, the ECOD method [32] adopts a nonparametric approach to estimate the intrinsic distribution of data, identifying anomalies as rare events that fall in the tails of the learned distribution. Deep-SVDD [33] integrates neural networks with the classical one-class support vector data description (SVDD) model, realizing end-to-end one-class anomaly detection. Building on the concept of classification-based anomaly detection, the GEOM [34] method proposes an image anomaly detection framework leveraging data augmentation techniques to enhance the generalization of the model. Further extending the GEOM method, the GOAD [34] method maps subspaces generated by random geometric transformations onto a hypersphere, then applies the one-class classification paradigm to achieve anomaly detection. However, a critical limitation of these classification-based and probability-based methods is that the geometric and random transformation strategies they rely on are difficult to directly adapt to industrial sensor time-series anomaly detection tasks. This is mainly because such transformations fail to account for the inherent temporal characteristics of time-series data (e.g., temporal order and long-range dependencies), making it challenging to capture the dynamic and sequential patterns of anomalies and cyberattacks in industrial sensor data.

### 2.2. Graph Neural Networks

In recent years, graph neural networks (GNNs) [11] have emerged as the de facto standard for graph analytics tasks owing to their powerful capability to model complex interrelationships between entities in graph-structured data. The core mechanism underlying GNNs is a message-passing scheme, which iteratively updates the feature embedding of each target node by aggregating and propagating the embeddings of its neighboring nodes. Specifically, representative GNN variants adopt distinct aggregation strategies: graph convolutional networks (GCNs) update node embeddings by assigning predefined weights to the messages (i.e., embeddings) transmitted from neighboring nodes; graph attention networks (GATs) adaptively learn the importance weights of each neighbor through an attention mechanism, followed by weighted aggregation to refine the target node’s representation; and graph isomorphism networks (GINs) leverage a universal aggregation function that can theoretically distinguish non-isomorphic graphs, updating node embeddings by aggregating neighbor features with learnable parameters to better capture the structural characteristics of graph data. As a representative GNN variant, GINs have shown remarkable performance in capturing fine-grained graph structural information, making them well suited for tasks involving complex graph structures, such as industrial sensor networks. Benefiting from their inherent ability to capture spatial dependencies and inter-entity correlations, GNNs (including GCNs, GATs, and GINs) have been widely adopted across various domains and applications—including industrial anomaly detection—where they effectively model the complex relationships between multiple sensors in industrial systems.

### 2.3. Semi-Supervised and Deep Learning Theory

Recent advancements in semi-supervised and foundational deep learning have introduced novel paradigms to address data scarcity and noisy real-world distributions. For instance, Fini et al. simplified semi-supervised learning via self-supervised clustering (e.g., SwAV/DINO), unifying representation learning and classification under a shared objective to achieve state-of-the-art results on large-scale vision benchmarks [35]. To mitigate confirmation bias caused by out-of-distribution noise, Yang et al.proposed class-aware contrastive semi-supervised learning (CCSSL), which decouples in-distribution and out-of-distribution samples for tailored regularization [36]. Beyond task-specific optimizations, Tian et al. provided a comprehensive survey on deep learning fundamentals, systematically reviewing architectural components and highlighting the necessity of modeling structured data [37]. Concurrently, in decentralized scenarios, Yang et al. introduced relation-guided aggregation for federated semi-supervised learning to prioritize reliable clients [38]. However, despite these significant strides, the existing paradigms predominantly treat sensors as independent entities or focus on image-based data, largely overlooking the inherent graph-structured physical dependencies within industrial sensor networks. This gap underscores the necessity of integrating graph learning with robust semi-supervised mechanisms for effective anomaly and cyberattack detection.

## 3. Methodology

This section elaborates on the detailed design of the proposed framework for industrial sensor anomaly and cyberattack detection. To ensure a clear and systematic presentation, the content is organized into three sections. Section 3.1 formally defines the task of industrial sensor anomaly and cyberattack detection, clarifying the input (sensor time-series data) and output (anomaly/attack identification) requirements. Section 3.2 presents the overall architecture of the dual-branch graph learning framework, including key components such as time-series patch segmentation, frequency-domain decomposition, Gaussian kernel-based dynamic graph learning (GSL), frequency gating (FreqGate), and gated recurrent unit (GRU) for temporal modeling. Section 3.3 introduces the loss function design, which guides the model training process and improves the accuracy of anomaly and attack detection.

### 3.1. Problem Definition

**Figure 1:**
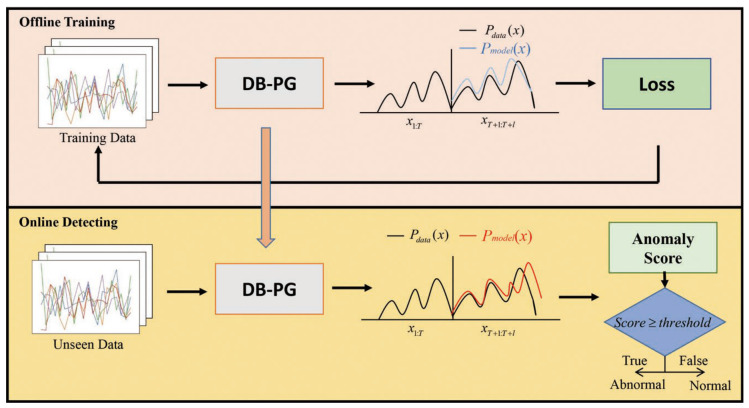
The workflow of the proposed DB-PG framework for industrial sensor anomaly and cyberattack detection, including offline training (model optimization on normal data) and online detection (anomaly identification via prediction error and threshold judgment).

This paper addresses multivariate time-series anomaly detection as an unsupervised learning task. The model is trained exclusively on anomaly-free data to predict the input series while minimizing prediction error. For normal inputs, predictions maintain high fidelity; however, anomalous inputs induce significant prediction deviations due to distributional shift. As illustrated in Figure 1, the proposed framework follows a prediction-based anomaly detection paradigm. During offline training, the DB-PG model is trained to fit the normal distribution of industrial sensor time series, minimizing the deviation between the model prediction $P_{\mathrm{model}}\left(x\right)$ and the real data $P_{\mathrm{data}}\left(x\right)$. During online detection, the model generates predictions for unseen data, and the anomaly score is calculated as the prediction error between the real and predicted values. A predefined threshold is then used to distinguish between normal operations and anomalies, including both cyberattacks and physical faults. This prediction-based paradigm enables the model to effectively detect unseen anomalies and cyberattacks without requiring labeled abnormal data, making it highly suitable for real-world industrial sensor systems.

Formally, let $X\in R^{N\times C}$ represent the multivariate time series with *N* time steps and *C* channels (sensors). Using sliding windows of length *w*, we define windowed series at time *t* as $X_{t}=\left\{x_{t-w+1},\ldots ,x_{t}\right\}\in R^{w\times C}$. The model $f:R^{w\times C}\to R^{l\times C}$ generates a prediction

(1) $${\hat{X}}_{t}=\left\{{\hat{x}}_{t+1},\ldots ,{\hat{x}}_{t+l}\right\}$$

(2) $${\hat{X}}_{t}=f\left(X_{t};\Omega \right)$$

where Ω is the set of learnable parameters of the model.

For an unseen test window $X_{t}^{test}=\left\{x_{t-w+1}^{test},\ldots ,x_{t}^{test}\right\}$, we predict its label $y_{t}\in 0,1$, where $y_{t}=1$ means the window is anomalous and $y_{t}=0$ means it is normal.

To further enhance model stability and performance, we employ a systematic data preprocessing pipeline with consistent robust normalization applied in both training and testing phases. Specifically, we adopt reversible instance normalization (RevIN) [10], a normalization approach tailored for time-series forecasting tasks. RevIN effectively mitigates distribution shift caused by dynamic variations in the mean and variance of temporal data, and its computational procedure is formulated as follows:

(3) $$\begin{array}{c} \mu =mean(X_{t})\\ \sigma =std(X_{t})\\ \bar{X_{t}}=\frac{X_{t}-\mu }{\sigma } \end{array}$$

where μ and σ represent the mean and standard deviation of $X_{t}$, respectively, and $\bar{X_{t}}$ denotes the windowed data after RevIN normalization.

### 3.2. Dual-Branch Patch Graph Block

#### 3.2.1. Overview Framework

**Figure 2:**
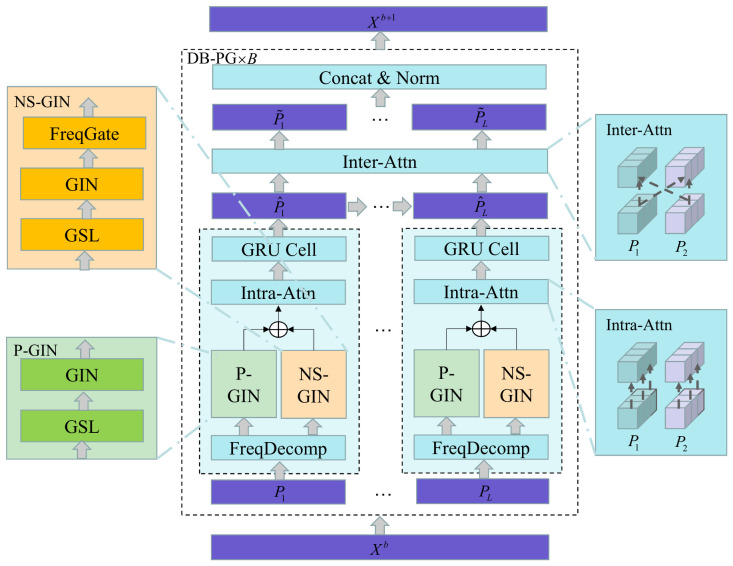
Architecture of the proposed DB-PG framework. The input time series is split into patches, decomposed into periodic and non-stationary components, and processed by dual GIN branches (P-GIN and NS-GIN) with dynamic graph learning. The NS-GIN branch is equipped with a FreqGate module to amplify abnormal features. Hierarchical temporal modeling (intra-attention, GRU, and inter-attention) is applied to capture local and global dependencies, producing the final output for anomaly detection.

The comprehensive overview of the proposed dual-branch patch graph (DB-PG) block framework is presented, with its overall architecture illustrated in Figure 2. The core design of DB-PG is to explicitly disentangle the periodic and non-stationary components of industrial sensor multivariate time series, and to model their spatial–temporal–frequency characteristics in a targeted manner, thereby achieving accurate anomaly and cyberattack detection.

As shown in Figure 2, given an input time-series batch $X^{b}$, the framework first segments the data into *L* overlapping patches $P_{1},P_{2},\ldots ,P_{L}$ to balance local feature extraction and computational efficiency. Each patch $P_{i}$ is then fed into a frequency decomposition (FreqDecomp) module, which decomposes the original time series into two distinct components:

- Periodic component: Captures the stable regular operational patterns of the industrial system, corresponding to normal working states;
- Non-stationary component: Contains high-frequency fluctuations, which are mainly derived from physical anomalies and malicious cyberattacks.

To model these two components separately, we design two dedicated graph learning branches, namely periodic GIN (P-GIN) and non-stationary GIN (NS-GIN):

- P-GIN Branch: This branch is dedicated to modeling the periodic component. It first employs a Gaussian kernel-based graph structure learning (GSL) module to adaptively learn the dynamic spatial dependencies between sensors, eliminating the need for predefined graph structures. The learned dynamic graph is then input into a graph isomorphism network (GIN) to extract high-level spatial features of the normal periodic patterns;
- NS-GIN Branch: This branch is designed for the non-stationary component. It follows the same GSL–GIN structure as P-GIN to model the spatial correlations of abnormal features and further integrates a frequency gating (FreqGate) module. FreqGate adaptively suppresses irrelevant low-frequency noise and amplifies critical high-frequency abnormal signals, significantly enhancing the model’s ability to capture subtle anomalies and attacks.

After extracting spatial features from both branches, the features are fused and input into an intra-attention (Intra-Attn) module to capture the fine-grained temporal dependencies within each patch. The output is then fed into a gated recurrent unit (GRU) cell to model the temporal evolution of features across time steps, generating the patch-level hidden representation $\hat{P_{i}}$ for each patch $P_{i}$.

To capture the long-range global temporal dependencies across different patches, all patch-level representations $\hat{P_{1}},\ldots ,\hat{P_{L}}$ are input into an inter-attention (Inter-Attn) module, which models the inter-patch correlations and generates the refined global patch representations $\tilde{P_{1}},\ldots ,\tilde{P_{L}}$. Finally, these global representations are concatenated to form the output $X^{b+1}$, which is used for subsequent prediction and anomaly scoring.

#### 3.2.2. Overlapping Patch Segmentation Strategy

To effectively capture the continuous temporal characteristics of industrial sensor time series and mitigate the information loss caused by patch boundary truncation, this paper adopts an overlapping patch segmentation strategy to process the input multivariate time-series data.

Given an input time-series batch $X^{b}\in R^{B\times C\times w}$, where *B* denotes the batch size, we first define the patch length $L_{p}$ and the sliding step size *s*. To balance temporal continuity and computational efficiency, we set a 50% overlap ratio between adjacent patches, i.e., $s=L_{p}/2$. The number of generated patches *L* is calculated as:

(4) $$L=\left\lfloor \frac{T-L_{p}}{s}\right\rfloor +1$$

Each patch $P_{i}\in R^{B\times C\times L_{p}}\left(i=1,2,\ldots ,L\right)$ is extracted from the original time series via a sliding window, where the *i*-th patch covers the time steps from (i−1)s+ to $\left(i-1\right)s+L_{p}$. The segmented overlapping patches are then fed into the FreqDecomp module for frequency-domain decomposition, laying a solid foundation for the subsequent dual-branch graph learning and hierarchical temporal modeling.

#### 3.2.3. Frequency-Domain Decomposition Module

To effectively disentangle the normal periodic patterns and abnormal non-stationary fluctuations in industrial sensor time series, this paper designs a two-stage frequency-domain decomposition (FreqDecomp) module, which sequentially performs seasonal-trend decomposition and Fourier spectral analysis to separate the input data into periodic and non-stationary components for subsequent dual-branch processing.

Seasonal-Trend Decomposition: Given an input time-series patch $P_{i}$, we first apply seasonal-trend decomposition to split the original signal into two interpretable components,

(5) $$\begin{array}{c} X_{trend}^{b}=AvgPool\left(Padding\left(X^{b}\right)\right)\\ X_{seasonal}^{b}=X^{b}-X_{trend}^{b} \end{array}$$

where

- $X_{seasonal}^{b}$ denotes the seasonal component, which captures the regular periodic operational patterns of the industrial system (corresponding to normal working states);
- $X_{trend}^{b}$ denotes the trend component, which contains the non-stationary aperiodic fluctuations, including physical anomalies, sensor drift, and malicious cyberattacks.

This decomposition effectively separates the stable baseline patterns from the dynamic abnormal variations, laying the foundation for targeted feature extraction in the subsequent dual-branch network. It is worth noting that the AvgPool operation here is not intended to provide a precise mathematical model of the trend; rather, it serves as a linear low-pass filter to coarsely separate the extremely slow-varying baseline from the raw signal. The intricate nonlinear characteristics and abrupt anomalies contained within the trend component are subsequently captured by the deep nonlinear architecture of the NS-GIN branch.

It is important to note that the seasonal-trend decomposition and the subsequent FFT-based filtering are not redundant operations but rather a coarse-to-fine refinement process. While seasonal-trend decomposition separates the signal into physically interpretable modes (stable periodicity vs. non-stationary trends), the FFT stage further purifies the periodic component by retaining only the top-k dominant frequency amplitudes. This prevents the model from overfitting to transient noise within the seasonal component and forces the periodic branch to focus on the fundamental operational cycles of the industrial system.

Fourier Spectral Analysis and Component Separation: To further refine the periodic component and extract the most representative normal patterns, we perform Fourier transform on the seasonal component $X_{seasonal}^{b}$ to obtain its frequency-domain representation

(6) $${\hat{X}}_{seasonal}^{b}=F\left(X_{seasonal}^{b}\right)$$

where F(·) denotes the fast Fourier transform (FFT) operation. We then calculate the amplitude spectrum of ${\hat{X}}_{seasonal}^{b}$ and select the top-*k* frequency components with the highest amplitudes, which correspond to the dominant periodic patterns of the industrial system. These components are inverse Fourier transformed to reconstruct the refined periodic component ${\tilde{X}}_{seasonal}^{b}$

(7) $${\tilde{X}}_{seasonal}^{b}=F^{-1}\left(\mathrm{Top}\text{-}k(|{\hat{X}}_{seasonal}^{b}|\right)$$

where Top-k(·) denotes the operation of retaining the top-*k* amplitude components and zeroing out the remaining frequencies.

Finally, we assign the two components to the corresponding branches of the graph framework:

- The refined periodic component ${\tilde{X}}_{seasonal}^{b}$ is fed into the P-GIN branch, which is dedicated to modeling normal periodic patterns and predicting normal sensor readings;
- The trend component $X_{trend}^{b}$ is directly input to the NS-GIN branch, which focuses on capturing non-stationary abnormal features and detecting anomalies and cyberattacks.

#### 3.2.4. Dual-Branch Graph Learning Network

Following the frequency-domain decomposition module, this section elaborates on the core dual-branch graph learning network of the proposed DB-PG framework, which consists of two dedicated branches: the periodic GIN (P-GIN) branch for normal pattern modeling and the non-stationary GIN (NS-GIN) branch for abnormal feature enhancement. The dual-branch design enables the model to explicitly disentangle and process periodic normal components and non-stationary abnormal components in a targeted manner, significantly improving the accuracy of industrial sensor anomaly and cyberattack detection.

Periodic GIN (P-GIN) Branch: The P-GIN branch is designed to model the refined periodic component ${\tilde{X}}_{seasonal}^{b}$ output by the FreqDecomp module, which contains the dominant normal operational patterns of industrial sensors. The branch consists of two core components: 1. graph structure learning (GSL) and 2. graph isomorphism network (GIN).

1. Graph Structure Learning (GSL)

To adaptively capture the dynamic spatial dependencies between sensors without relying on predefined graph structures, we first employ a Gaussian kernel-based GSL module to generate the dynamic adjacency matrix $A_{i}^{P}$ for the periodic component ${\tilde{X}}_{seasonal}^{b}$. Given the feature matrix of the periodic component ${\tilde{X}}_{seasonal}^{b}$, the similarity between sensor *u* and sensor *v* is calculated as

(8) $$A_{i,uv}^{P}=\exp\left(-\frac{\left|\right|{\tilde{X}}_{seasonal,u}^{b}-{\tilde{X}}_{seasonal,v}^{b}{\left|\right|}_{2}^{2}}{2\sigma^{2}}\right)$$

where σ is the kernel bandwidth parameter, which controls the sensitivity of the similarity calculation. The generated adjacency matrix $A_{i}^{P}$ dynamically reflects the spatial correlation between sensors under normal operating conditions, laying a foundation for accurate graph feature extraction. Crucially, the Gaussian kernel is employed to capture instantaneous physical couplings between sensors—such as hydraulic pressure propagation—rather than to infer strict causal directions. Despite being a shallow similarity measure, it provides a stable and differentiable foundation for dynamic graph generation. Subsequently, the graph isomorphism network (GIN) reasons over this estimated topology to extract high-level interaction patterns.

2. Graph Isomorphism Network (GIN)

We then feed the periodic component ${\tilde{X}}_{seasonal}^{b}$ and the dynamic adjacency matrix $A_{i}^{P}$ into the GIN to extract high-level spatial features of normal patterns. The GIN is a powerful graph neural network that can distinguish different graph structures and capture fine-grained node features, with the update formula for the *l*-th layer defined as

(9) $$h_{v}^{\left(l+1\right)}=\mathrm{MLP}\left(\left(1+\varepsilon^{\left(l\right)}\right)h_{v}^{\left(l\right)}+\sum_{u\in N(v)}h_{u}^{\left(l\right)}\right)$$

where $h_{v}^{\left(l\right)}$ is the feature of node *v* at the *l*-th layer, $\varepsilon^{\left(l\right)}$ is a learnable parameter, and N(v) is the set of neighbors of node *v*. The GIN effectively aggregates the spatial features of sensors, enabling the P-GIN branch to learn the complete normal operational pattern of the industrial system.

The output feature of the P-GIN branch $H_{P,i}\in R^{N\times d^{\prime }}$ is then fused with the output of the NS-GIN branch for subsequent temporal modeling.

Non-Stationary GIN (NS-GIN) Branch: The NS-GIN branch is dedicated to processing the trend component $X_{trend}^{b}$ output by the FreqDecomp module, which contains non-stationary fluctuations caused by physical anomalies, sensor drift, and malicious cyberattacks. On the basis of the same GSL–GIN structure as the P-GIN branch, the NS-GIN branch further integrates a frequency gating (FreqGate) module to enhance the extraction of abnormal features.

1. GSL and GIN for Non-Stationary Components

Similar to the P-GIN branch, we first use the Gaussian kernel-based GSL module to generate the dynamic adjacency matrix $A_{i}^{NS}$ for the trend component $X_{trend}^{b}$, which captures the spatial correlation of sensors under abnormal conditions. The trend component $X_{trend}^{b}$ and the adjacency matrix $A_{i}^{NS}$ are then input into the GIN to extract the spatial features of non-stationary components, obtaining the initial feature $H_{NS,i}^{\prime }\in R^{N\times d^{\prime }}$.

2. Frequency Gating (FreqGate) Module

To further amplify the high-frequency abnormal signals and suppress the low-frequency normal noise in the non-stationary component, we design the FreqGate module to adaptively weight the features output by the GIN. The FreqGate module first performs Fourier transform on the feature $H_{NS,i}^{\prime }$ to obtain its frequency-domain representation, then calculates the amplitude spectrum to generate the frequency attention weight $W_{f}$, and finally applies the weight to the original feature

(10) $$H_{NS,i}=F^{-1}\left(F\left(H_{NS,i}^{\prime }\right)⊙W_{f}\right)$$

where ⊙ is the element-wise multiplication, σ(·) is the sigmoid activation function, and $W_{f}$ is the learnable frequency attention weight, which is adaptively updated during the model training process. The FreqGate module significantly enhances the model’s sensitivity to subtle anomalies and cyberattacks, effectively improving the detection performance of the NS-GIN branch. The frequency attention weight $W_{f}$ is not predefined; instead, it is generated by a learnable multilayer perceptron (MLP) that takes the amplitude spectrum as input. This allows the model to autonomously identify and emphasize frequency bands most indicative of anomalies, adapting to specific industrial dynamics.

Dual-Branch Feature Fusion: After extracting spatial features from the P-GIN and NS-GIN branches, respectively, we fuse the two branch features to integrate normal pattern information and abnormal feature information. The fusion operation is defined as

(11) $$H_{i}=H_{P,i}+H_{NS,i}$$

where $H_{i}\in R^{N\times d^{\prime }}$ is the fused patch-level spatial feature, which is then input into the subsequent intra-attention and GRU modules for temporal modeling.

The dual-branch graph learning network effectively realizes the targeted modeling of normal and abnormal components, making the model not only accurately fit the normal operational patterns of industrial sensors but also highly sensitive to various anomalies and cyberattacks, laying a solid foundation for the overall performance of the DB-PG framework.

#### 3.2.5. Temporal Modeling with Intra-Attn, GRU and Inter-Patch Attention

Following the dual-branch graph feature fusion, we adopt a hierarchical temporal modeling pipeline to capture both local fine-grained temporal dependencies and global long-range correlations, which consists of intra-patch attention (Intra-Attn), gated recurrent unit (GRU), and inter-patch attention (Inter-Attn). The pipeline strictly follows the logical order of “intra-patch, patch-level temporal evolution, inter-patch global aggregation”, ensuring the model can fully learn the temporal characteristics of industrial sensor time series.

Intra-Patch Attention: To capture fine-grained temporal dependencies within each patch, we employ non-causal self-attention for Intra-Attn, which allows each time step to attend to all forward and backward time steps within the patch. This is critical for identifying local anomalous outliers that require full contextual information.

We first project $H_{i}$ into query, key, and value matrices using learnable projection matrices $W_{Q},W_{K},W_{V}$:

(12) $$Z_{i}=\mathrm{MLP}(H_{i})$$

(13) $$Q_{i}=Z_{i}W_{Q},K_{i}=Z_{i}W_{K},V_{i}=Z_{i}W_{V}$$

The non-causal attention weight matrix $M_{intra,i}$ is computed via scaled dot-product attention without any causal masking:

(14) $$M_{intra,i}=\mathrm{softmax}\left(\frac{Q_{i}K_{i}^{T}}{\sqrt{d}}\right)$$

The enhanced intra-patch temporal feature ${\tilde{Z}}_{i}$ is obtained by weighting the value matrix with the attention weights:

(15) $${\tilde{Z}}_{i}=M_{intra,i}V_{i}$$

To stabilize model training and preserve original feature information, we add a residual connection and layer normalization:

(16) $${\tilde{Z}}_{i}=\mathrm{LayerNorm}\left(Z_{i}+{\tilde{Z}}_{i}\right)$$

GRU for Patch-Level Temporal Evolution: After obtaining the enhanced intra-patch feature ${\tilde{Z}}_{i}$ (output by the Intra-Attn module), we treat the entire patch as a single input step to the GRU layer rather than splitting it into individual time steps. This design aligns with the patch-wise processing paradigm of the DB-PG framework, focusing on modeling the temporal evolution between patches. The update rule of the GRU is redefined to fit the patch-level input

(17) $${\hat{P}}_{i}=\mathrm{GRU}\left({\hat{P}}_{i-1},{\tilde{Z}}_{i}\right)$$

where ${\hat{P}}_{i}$ denotes the hidden state output by the GRU after processing the *i*-th patch.

This design ensures that the GRU focuses on learning the temporal transition relationship across consecutive patches rather than redundant modeling of within-patch time steps. After processing all *L*, we collect the hidden states of all patches ${\hat{P}}_{1},\ldots ,{\hat{P}}_{L}$ to form the patch-level temporal representation sequence $P=[{\hat{P}}_{1},\ldots ,{\hat{P}}_{L}]$, which is then fed into the Inter-Attn module for global long-range dependency modeling.

Inter-Patch Attention for Global Dependencies: After obtaining the fused spatial–temporal features, we adopt inter-patch attention to capture long-range global dependencies, with temporal masking to ensure full contextual interaction. Consistent with the intra-patch attention structure, the inter-patch attention does not repeat the redundant formula derivation and directly outputs the globally refined representation ${\tilde{P}}_{i}$.

(18) $${\tilde{P}}_{1},\ldots ,{\tilde{P}}_{L}=\mathrm{LayerNorm}\left(P+Attn^{causal}\left(P\right)\right)$$

Following the inter-patch attention module, we concatenate the refined patch-level representations $\left\{{\tilde{P}}_{1},\ldots ,{\tilde{P}}_{L}\right\}$ along the temporal axis and apply layer normalization to obtain the input of the next DB-PG block:

(19) $$X^{b+1}=\mathrm{LayerNorm}\left(Concat({\tilde{P}}_{1},\ldots ,{\tilde{P}}_{L})\right)$$

The output $X^{B}$ of the last DB-PG block is used as the final prediction ${\hat{X}}_{t}$.

### 3.3. Loss Function and Anomaly Scoring

To effectively train the DB-PG framework and enhance its anomaly detection performance, we design a multi-component loss function, which integrates mean squared error (MSE) prediction loss, graph sparsity loss, graph smoothness loss, and structural similarity index measure (SSIM) loss. This combined loss guides the model to fit normal patterns, maintain reasonable graph structures, and improve feature representation quality, while the anomaly score is derived from the prediction deviation.

Multi-Component Loss Function: The total loss function $L_{total}$ is a weighted combination of the four components, defined as

(20) $$L_{total}=\lambda_{1}L_{MSE}+\lambda_{2}L_{sparse}+\lambda_{3}L_{smooth}+\lambda_{4}L_{ssim}$$

where $\lambda_{1},\lambda_{2},\lambda_{3},\lambda_{4}$ are weights that balance the contribution of each loss component.

1. MSE Prediction Loss

The MSE loss is used to minimize the deviation between the predicted time series ${\hat{X}}_{t}$ and the original input $X_{t}$. It is defined as:

(21) $$L_{\mathrm{MSE}}=\frac{1}{Cw}{∥X_{t}-{\hat{X}}_{t}∥}^{2}$$

2 Graph Sparsity Loss

To avoid redundant spatial dependencies and ensure the learned graph adjacency matrices $A_{i}^{P}$ and $A_{i}^{NS}$ are sparse (consistent with the sparse connection characteristics of industrial sensors), we introduce the graph sparsity loss, defined using the $L_{1}$ norm:

(22) $$L_{\mathrm{sparse}}=\frac{1}{LC^{2}}\sum_{i=1}^{L}\left({∥A_{i}^{P}∥}_{1}+{∥A_{i}^{NS}∥}_{1}\right)$$

This loss constrains most elements in the adjacency matrix to be zero, reducing computational complexity and enhancing the interpretability of the graph structure.

3. Graph Smoothness Loss

The graph smoothness loss ensures that spatially adjacent sensors have similar feature representations, which aligns with the physical correlations of industrial sensors. It is defined as

(23) $$\begin{array}{cc} L_{\mathrm{smooth}}= & \frac{1}{C}\sum_{i=1}^{L}(\mathrm{Tr}\left({H_{P}}^{⊤}D_{i}^{P}H_{P}\right)-\mathrm{Tr}\left({H_{P}}^{⊤}A_{i}^{P}H_{P}\right)\\ \; & +\mathrm{Tr}\left({H_{NS}}^{⊤}D_{i}^{NS}H_{NS}\right)-\mathrm{Tr}\left({H_{NS}}^{⊤}A_{i}^{NS}H_{NS}\right)) \end{array}$$

where *H* denotes the node feature matrix output by the dual-branch graph learning module, with each row $h_{u}^{T}$ representing the *d*-dimensional feature vector of sensor *u*. Tr(·) is trace operator, which sums the diagonal elements of a matrix and is used to compactly represent the sum of pairwise feature differences. *D* is the degree matrix of the graph. While seemingly contradictory, the sparsity and smoothness regularizers serve complementary roles: sparsity eliminates noisy or redundant edges to prevent overfitting, whereas smoothness ensures that the retained physical couplings evolve stably over time. This combination acts as a structural prior tailored for industrial sensor networks.

4. SSIM Loss

Compared with MSE, the SSIM loss [39] better captures the structural similarity between the original and predicted time series, focusing on texture and temporal continuity. It is computed as

(24) $$\begin{array}{c} L_{\mathrm{ssim}}=1-SSIM\left(X^{b},{\hat{X}}^{b}\right)\\ \mathrm{SSIM}\left(X^{b},{\hat{X}}^{b}\right)=\frac{\left(2\mu_{X^{b}}\mu_{{\hat{X}}^{b}}+C_{1}\right)\left(2\sigma_{X^{b}{\hat{X}}^{b}}+C_{2}\right)}{\left(\mu_{X^{b}}^{2}+\mu_{{\hat{X}}^{b}}^{2}+C_{1}\right)\left(\sigma_{X^{b}}^{2}+\sigma_{{\hat{X}}^{b}}^{2}+C_{2}\right)} \end{array}$$

where $SSIM(X^{b},{\hat{X}}^{b})$ denotes the structural similarity index between the original input $X^{b}$ and the predicted ${\hat{X}}^{b}$. Minimizing this loss ensures the predicted time series maintains the structural characteristics of normal data. $C_{1}$ and $C_{2}$ are small constants to avoid numerical instability, set as $C_{1}=0.0001$ and $C_{2}=0.0009$ following the standard SSIM configuration.

Anomaly Score: During inference, the anomaly score comprehensively considers the prediction error (MSE and SSIM) and the abrupt variation in the learned graph structure between consecutive patches. The former reflects the deviation of predicted values from normal patterns, while the latter captures sudden changes in spatial sensor correlations, which often indicate anomalies or cyberattacks. The final anomaly score is defined as

(25) $$S=\alpha_{1}\frac{1}{wC}{∥X^{b}-{\hat{X}}^{b}∥}^{2}+\alpha_{2}\mathrm{SSIM}\left(X^{b},{\hat{X}}^{b}\right)+\alpha_{3}\frac{1}{L-1}\sum_{l=1}^{L-1}\frac{\left|\right|A_{i}^{NS}-A_{i+1}^{NS}{\left|\right|}_{F}}{C^{2}}$$

where $\alpha_{i}$ is weight and $\left|\right|\cdot {\left|\right|}_{F}$ denotes the Frobenius norm. A higher anomaly score indicates either a large prediction deviation or an abrupt graph structure change, which corresponds to a higher probability of anomalous behavior. By setting an appropriate threshold on S, we can distinguish normal and abnormal samples for industrial sensor anomaly detection. It is worth noting that, while the graph structures are generated dynamically for each patch, the inherent continuity of the input time series naturally regularizes the evolution of these graphs under normal conditions. The anomaly scoring mechanism leverages deviations from this natural consistency rather than enforcing strict temporal smoothness on the graph topology itself.

## 4. Experiments and Discussion

In this section, we conduct experiments to answer the following research questions:

- RQ1 (Accuracy): Does our method outperform baseline methods in accuracy of anomaly detection in multivariate time series based on ground-truth-labeled anomalies?
- RQ2 (Ablation): How do the various components of the method contribute to its performance?
- RQ3 (Comprehensiveness): Can our approach detect all types of network attacks?
- RQ4 (Visualization): Can visualization methods be used to explain how anomalies are detected?

### 4.1. Datasets

Given the scarcity of real-world labeled anomaly datasets, especially for large-scale industrial plants, we employ four sensor datasets collected from water treatment physical testbeds: SWaT, WADI [11], HAI [40] and BATADAL [41]. In all the datasets, operators simulate realistic attack scenarios similar to those encountered in actual water treatment facilities, with the corresponding events carefully labeled as ground-truth anomalies. Table 1 summarises the statistics of the four datasets.

The Secure Water Treatment (SWaT) dataset is collected from a water treatment testbed operated by Singapore’s Public Utilities Board. It represents a scaled-down yet representative cyber–physical system that integrates digital and physical components to monitor and control system operations. As an extension of SWaT, the Water Distribution (WADI) dataset covers a more extensive water distribution network, offering a more comprehensive and realistic characterization of a full-scale water treatment, storage, and distribution system. The HIL-based augmented ICS (HAI) dataset is collected from a complex hardware-in-the-loop testbed developed by the Korea Atomic Energy Research Institute (KAERI). It represents a highly integrated cyber–physical system that couples multiple physical processes, including boiler, turbine, and water treatment units, to simulate complex industrial interactions. As a variant focusing on infrastructure-scale simulations, the BATADAL (BATtle of the Attack Detection Algorithms) dataset covers a large-scale water distribution network, offering a comprehensive benchmark for detecting stealthy cyberattacks in hydraulic systems.

### 4.2. Metrics

We employ precision (*Pre*), recall (*Rec*), and F1-score (*F*1) metrics over the test dataset and its corresponding ground-truth values to assess the performance of our method and baseline models,

(26) $$\begin{array}{c} Pre=\frac{TP}{TP+FP} \end{array}$$

(27) $$\begin{array}{c} Rec=\frac{TP}{TP+FN} \end{array}$$

(28) $$\begin{array}{c} F1=\frac{2\times Pre\times Rec}{Pre+Rec} \end{array}$$

where *TP*, *TN*, *FP*, and *FN* represent the counts of true positives, true negatives, false positives, and false negatives. For anomaly detection, we determine the threshold by considering the maximum anomaly score across the validation dataset. During testing, any time step with an anomaly score exceeding the threshold α is classified as an anomaly. Notably, these metrics are calculated using the point adjustment strategy [10], in line with the approach used in most previous studies [10]. The point adjustment strategy is well suited for anomaly detection in industrial control scenarios since anomalous events typically persist over multiple consecutive time steps.

### 4.3. Baselines

We compare our DB-PG with a wide range of state-of-the-art approaches in multivariate time-series anomaly detection, including:

1. MAD-GAN, an anomaly detection algorithm based on GAN network.
2. OmniAnomaly, a deep learning-based algorithm that uses a combination of autoencoders and CNNs.
3. ModernTCN, an anomaly detection approach based on CNN.
4. MTAD-GAT, a GNN anomaly detection algorithm based on prediction and reconstruction methods.
5. GDN, an anomaly detection approach based on GNN single-point prediction.
6. TranAD, a multimodal anomaly detection algorithm based on transformer.
7. Anomaly transformer, an anomaly detection algorithm based on transformer.
8. FED, a transformer anomaly detection algorithm based on Fourier transform.
9. USAD, a method via adversarially trained AEs.
10. TSMixer, a lightweight prediction algorithm based on MLP.
11. DCdetector, an anomaly detection algorithm based on dual attention contrastive representation learning.
12. MODEM, a diffusion model-based time-series anomaly detection framework that incorporates spectral domain analysis into its architecture.
13. ImDiffusion, a diffusion model incorporating an imputation mechanism for time-series anomaly detection.
14. CATCH, a multi-band multi-channel time-series anomaly detection model.

### 4.4. Implementation Details

We implemented our method and baselines using PyTorch version 2.0.0, with CUDA 11.7 and the PyTorch Geometric Library version 1.5.0. The training was conducted on a Ubuntu server with Intel® Xeon® CPU E5-2640 @ 2.50GHz and an NVIDIA 4090 GPU. Consistent with an unsupervised learning paradigm, the model is fitted exclusively on unlabeled normal data. Anomalies are detected at inference time by comparing the calculated anomaly score against a fixed threshold; scores exceeding this threshold indicate abnormal time stamps. The relevant hyperparameters are provided in Table 2.

### 4.5. RQ1. Accuracy

As shown in Table 3 and Table 4, we report the anomaly detection performance of the proposed DB-PG and baseline methods in terms of precision, recall, and F1-score on two datasets. As observed from the results, DB-PG achieves state-of-the-art performance under the widely used F1-score metric. Moreover, it yields the highest or second-highest precision and recall values on most datasets. This demonstrates DB-PG’s robust balance between false positive and true positive rates across multiple decision thresholds, which is crucial for real-world deployment, especially in industrial control systems.

Further observations can be summarized as follows:

1. Traditional anomaly detection methods based on VAE and GAN architectures, such as MAD-GAN, exhibit inferior performance compared with recent approaches. This is mainly due to their insufficient modeling of inter-sensor correlations and intra-series temporal dependencies, as well as their failure to capture key properties such as periodicity in time series;
2. Compared with other GNN-based models, including GDN and MTAD-GAT, DB-PG achieves more accurate detection results. This improvement mainly comes from DB-PG’s ability to model multi-periodicity and its multi-scale graph convolution mechanism, which effectively captures richer temporal patterns. In contrast, GDN and MTAD-GAT only focus on temporal features while ignoring the spectral characteristics of time series;
3. Unlike transformer-based methods such as anomaly transformer, FED, and TranAD, DB-PG pays more attention to trend components and explicitly models frequency-domain representations;
4. For recent diffusion-based detection models such as MODEM and ImDiffusion, their inherent insensitivity to high-frequency components leads to unstable performance near event boundaries, resulting in missed detections at the start and end of anomalous events.

Comparative experiments confirm that, in industrial control time series, periodic components represent statistically dominant features, and residual trend components are also non-negligible. Meanwhile, the frequency domain provides a more discriminative representation for identifying anomalous behaviors.

### 4.6. RQ2. Ablation

To validate the efficacy of each core component in the proposed DB-PG framework, we conduct a systematic ablation study on the SWaT, WADI, HAI and BATADAL datasets. The corresponding quantitative results are presented as follows. The detailed ablation results on the public benchmarks are illustrated in Table 5 and Table 6, respectively.

As observed, eliminating either the periodic or trend branch from the dual-branch architecture leads to considerable performance deterioration, indicating that the joint modeling of periodic and trend components is indispensable for capturing comprehensive temporal characteristics. Removing the frequency decomposition module and adopting a single-branch structure with raw patch input also results in notable performance decline, which verifies that frequency-aware disentanglement facilitates the extraction of discriminative representations for industrial anomaly detection.

Moreover, substituting the adaptive graph learning module with a fixed fully connected graph yields inferior detection performance, demonstrating that data-driven adaptive topology modeling enables more accurate and reasonable capture of underlying spatial dependencies among sensors. Furthermore, removing the frequency gating mechanism gives rise to a substantial drop in overall scores, confirming its effectiveness in emphasizing spectrally salient features associated with anomalous events.

Furthermore, we investigate the impact of the graph variation term in the anomaly scoring function. As shown in Table 5 and Table 6, removing this term (w/o graph variation) leads to a noticeable decrease in detection accuracy. This validates that monitoring the dynamic evolution of the graph structure provides complementary information to prediction errors. While prediction errors reflect deviations in sensor readings, graph variations capture the disruption of underlying spatial dependencies, which is particularly crucial for detecting sophisticated cyberattacks that manipulate multiple sensors. Ablation studies in Table 5 and Table 6 confirm that GIN outperforms GCN because its sum-based injective aggregation preserves richer structural information and maintains higher stability under non-stationary industrial conditions.

Finally, the exclusion of either intra-patch or inter-patch attention impairs model performance. In particular, the ablation of intra-patch attention incurs a more significant performance reduction, suggesting that modeling local temporal dependencies within individual patches plays a more critical role in identifying subtle and fine-grained anomalies. In conclusion, all the proposed modules collectively contribute to the final detection performance, and their integration enables the superior robustness and accuracy of DB-PG in industrial time-series anomaly detection.

### 4.7. RQ3. Comprehensiveness

Our experiments benchmark the proposed DB-PG against state-of-the-art attack detection methods on the SWaT, WADI, HAI and BATADAL industrial control datasets. Detection performance is evaluated based on the number of successfully identified attacks, where SWaT contains 36 distinct attack scenarios, WADI contains 14 attacks (note that Attacks 3 and 4 in WADI are merged into a single attack type), HAI encompasses 58 attack scenarios, and BATADAL comprises 14 distinct attack cases.

As shown in Table 7, DB-PG achieves superior cyberattack detection performance compared with all the baseline methods, especially demonstrating stronger ability in capturing temporal propagation patterns embedded in time-series data.

To further illustrate the attack detection effectiveness of DB-PG in detail, Table 8 reports the precision, recall, and F1-score of DB-PG for each stage of the SWaT dataset. In Table 8, successfully detected attacks are underlined, and attacks targeting devices across multiple stages (e.g., Attacks 22 and 23) are repeated in their corresponding rows. It can be observed that DB-PG achieves outstanding detection performance over various attack types distributed across the six stages.

Table 9, Table 10 and Table 11 present the precision, recall, and F1-scores attained by DB-PG for individual attacks across the WADI, HAI, and BATADAL datasets. As demonstrated, the proposed DB-PG method consistently exhibits robust and reliable detection performance across all the attack types within all the datasets.

### 4.8. RQ4. Visualization

#### 4.8.1. Visual Analysis of Trend-Aware Graph Evolution

**Figure 3:**
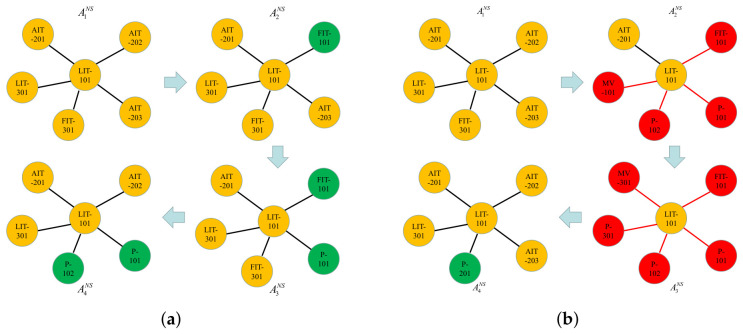
Visualization of trend-aware graph evolution under normal and attack scenarios in the SWaT dataset. (**a**) Under normal operation, the trend graph centered at LIT-101 evolves smoothly with stable sensor correlations. (**b**) Under Attack 21, the graph structure undergoes abrupt topological changes due to the manipulated trend of LIT-101, indicating system anomalies.Yellow nodes denote sensors with stable long-term physical correlations. Green nodes denote actuators and flow sensors exhibiting dynamic correlations. Red nodes denote sensors whose temporal correlation with LIT-101 is severely disrupted.

To further interpret the anomaly detection mechanism of the proposed DB-PG framework, we conduct a visual analysis of the trend-aware graph adjacency matrix evolution under normal and attack conditions, as illustrated in Figure 3. This visualization intuitively demonstrates how the dynamic topological structure of sensor correlations responds to system anomalies, providing interpretable evidence for the effectiveness of trend-aware graph learning. As shown in subfigure (a), under normal operating conditions, the trend graph centered on the core sensor LIT-101 (raw water tank level) exhibits smooth gradual structural changes across consecutive patches ($A_{1}^{NS}$ to $A_{4}^{NS}$). Yellow nodes represent sensors with stable long-term physical correlations with LIT-101 (e.g., AIT-201/202/203, LIT-301, and FIT-301), maintaining consistent connectivity with the central node. Green nodes denote dynamically correlated actuators and flow sensors (e.g., FIT-101, P-101, and P-102) that join or leave the strong correlation set in an orderly manner, reflecting the healthy predictable dependency relationships of the industrial system in normal operation. In contrast, subfigure (b) presents the trend graph evolution under a typical cyberattack (Attack 21 on the SWaT dataset), where LIT-101 is maliciously manipulated to exhibit an unnatural rising trend. A drastic abrupt structural mutation occurs across patches $A_{1}^{NS}$ to $A_{4}^{NS}$: red nodes indicate sensors whose temporal correlations with LIT-101 are severely disrupted (e.g., FIT-101, MV-101, P-101, and P-102). The forced anomalous trend of LIT-101 breaks inherent physical dependencies, causing a sudden collapse of the original stable graph structure. This significant topological change directly reflects system anomalies, enabling DB-PG to detect attacks by monitoring deviations from normal graph evolution patterns. The comparative visualization verifies two key advantages of DB-PG: (1) the trend-aware graph learning module effectively captures dynamic temporal dependencies among sensors, maintaining stability under normal conditions and responding sensitively to attack-induced structural mutations; (2) the dynamic trend graph evolution provides interpretable evidence for anomaly detection, transforming black-box deep learning predictions into visually explainable topological changes—critical for practical deployment in industrial control systems. This analysis further confirms the indispensable role of the trend branch in the dual-branch architecture for detecting cyberattack-induced structural anomalies.

#### 4.8.2. Visual Validation of Joint MSE–SSIM Prediction Loss

**Figure 4:**
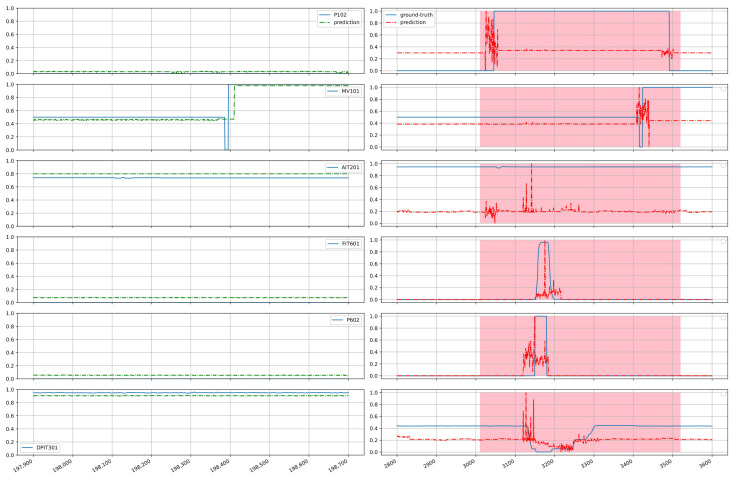
(**Left**): Under normal operation, the predicted values (green dashed lines) closely align with the ground truth (blue solid lines), verifying the high prediction accuracy of the model. (**Right**): Under cyberattack (pink shaded area), the predicted values (red dashed lines) exhibit immediate abnormal fluctuations at the attacked sensor (P102) and delayed responses at other correlated sensors, demonstrating the model’s ability to capture sensor dependencies and anomaly propagation.

To provide a more intuitive illustration of the interactions between correlated sensors, we plot the time-series variations in the top five sensors with the strongest correlations under normal and attack conditions, as shown in Figure 4.

In the left panel, under normal operating conditions, the predicted values of the sensors (green dashed lines) closely follow the true data (blue solid lines), demonstrating the high prediction accuracy of the proposed DB-PG framework enabled by the joint MSE–SSIM loss. When an anomaly occurs (e.g., P102 is suddenly activated at 3000 s and deactivated at 3500 s, as shown in the right panel), the predicted values exhibit significant abnormal fluctuations. The prediction of P102 changes instantaneously, while the predictions of other correlated sensors show delayed responses, which visually reflects the propagation of anomalies in the sensor network.

Figure 4 effectively validates that DB-PG can accurately learn the dynamic dependencies among industrial sensors, laying a solid foundation for reliable anomaly detection.

#### 4.8.3. Frequency-Domain Characterization of Normal and Abnormal Signals

**Figure 5:**
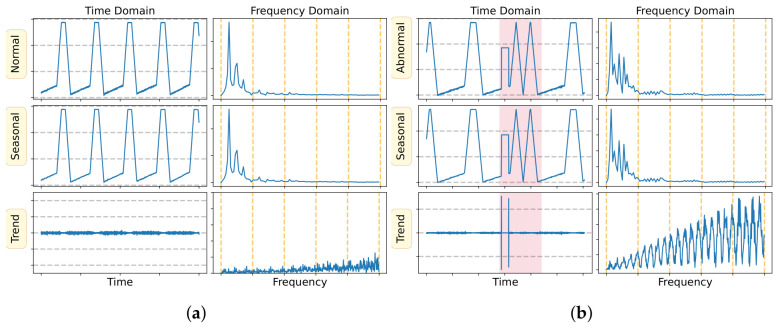
Time-domain and frequency-domain visualization of LIT-101 sensor data, illustrating the motivation for the frequency-domain gating mechanism. (**a**) Under normal operation, the signal features stable periodic patterns with low-frequency energy concentration. (**b**) Under cyberattack, the periodic waveform is disrupted, and the trend component exhibits severe temporal mutations accompanied by a significant surge in full-band frequency energy, which the frequency-domain gating mechanism amplifies to enhance anomaly detection performance. The pink shaded area denotes the time period during which the system was under attack.

To explicitly validate the effectiveness of the proposed frequency-domain gating (FreqGate) mechanism in the DB-PG framework, we conduct a time–frequency analysis of the core sensor LIT-101 under normal and attack conditions, as illustrated in Figure 5. This analysis directly demonstrates how FreqGate amplifies discriminative anomaly features while suppressing redundant normal components, laying a solid foundation for high-performance anomaly detection.

As shown in Figure 5a, under normal operating conditions, the raw LIT-101 signal exhibits stable regular periodic fluctuations in the time domain. The seasonal component accurately captures the inherent cyclic patterns of the raw water tank level, while the trend component remains smooth and steady, reflecting the system’s healthy steady-state operation. In the frequency domain, the spectral energy of both the raw signal and the seasonal component is highly concentrated in the low-frequency band, which corresponds to the normal periodic operation of the industrial water treatment system. The trend component, by contrast, shows only weak uniformly distributed noise across the full frequency band, with no significant energy peaks.

In stark contrast, Figure 5b presents the signal characteristics under a typical cyberattack (Attack 21 on the SWaT dataset), where LIT-101 is maliciously manipulated to exhibit an unnatural rising trend. The original periodic waveform is severely distorted, with abrupt mutations occurring within the attack interval (marked by the pink shaded area). Most critically, the trend component undergoes dramatic temporal anomalies, and its frequency spectrum exhibits a striking surge of high-amplitude energy across the entire frequency band—an obvious deviation from the normal state.

This stark frequency-domain discrepancy between normal and abnormal signals is the core motivation for the FreqGate mechanism. FreqGate is designed to dynamically weight frequency components based on their anomaly sensitivity: it suppresses the stable low-frequency energy that dominates normal signals while significantly amplifying the abnormal high-frequency energy that emerges exclusively during attacks. As visualized in Figure 5, the trend component, which is most sensitive to structural anomalies, exhibits the most pronounced frequency-domain difference between normal and attack states. FreqGate leverages this property to enhance the discriminability of anomaly features in the trend branch, enabling the model to detect subtle structural anomalies caused by cyberattacks with higher sensitivity and accuracy.

In summary, this time–frequency analysis verifies two key advantages of the proposed FreqGate mechanism: (1) it effectively exploits the inherent frequency differences between normal and abnormal signals, amplifying critical anomaly features while suppressing redundant normal components; (2) it significantly enhances the anomaly detection capability of the trend branch in the dual-branch architecture, making DB-PG highly robust to various cyberattacks in industrial control systems.

#### 4.8.4. Hyperparameter Sensitivity

To evaluate the sensitivity of the proposed DB-PG model to its core hyperparameters, we conducted a grid search on the SWaT dataset by varying the FFT top-k value ($f_{k}$) and the seasonal decomposition window size ($S_{w}$). As illustrated in Table 12, the model achieves its peak performance (F1-score: 96.58%) when $f_{k}=5$ and $S_{w}=13$.

The results reveal distinct trends: (1) Impact of $f_{k}$: The performance initially increases with $f_{k}$, reaches a plateau around $f_{k}=5$, and then gradually declines. This suggests that excessively low $f_{k}$ fails to capture sufficient periodic information, while excessively high $f_{k}$ introduces high-frequency noise that hinders detection. (2) Impact of $S_{w}$: The model exhibits relatively stable performance for $S_{w}$ values ranging from 11 to 15. However, extreme values (e.g., $S_{w}=7$ or $S_{w}=17$) lead to noticeable degradation. This indicates that the seasonal window must be appropriately sized to match the intrinsic cycle length of the industrial process; otherwise, it risks underfitting or overfitting the trend component.

#### 4.8.5. Complexity and Real-Time Performance Analysis

**Figure 6:**
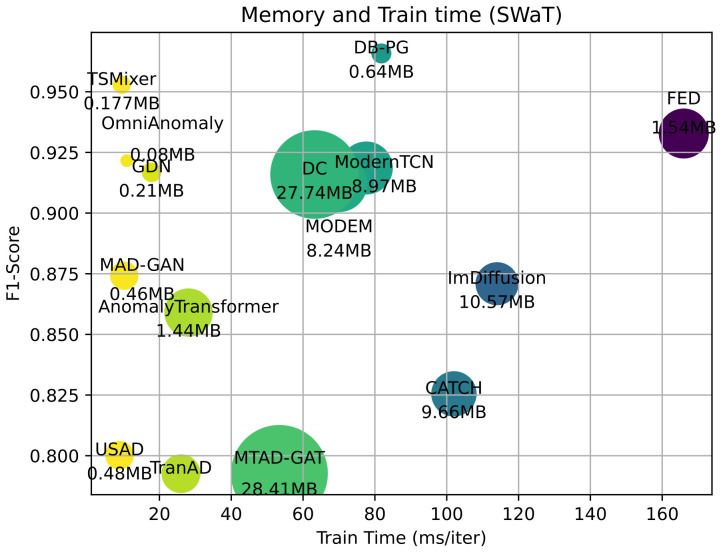
Memory Consumption and Training Time on the SWaT Dataset.

To evaluate the practicality of the proposed DB-PG framework for industrial deployment, we analyze its computational complexity and resource consumption on the SWaT dataset. As illustrated in Figure 6, DB-PG achieves exceptional efficiency, requiring only 0.64 ms per training iteration and a minimal memory footprint of 0.64 MB.

Compared to the baseline methods, DB-PG demonstrates superior lightweight characteristics. While transformer-based architectures like MAD-GAN consume 10.57 MB of memory, our method reduces memory usage by approximately 94% while maintaining higher detection accuracy. Although lightweight models like USAD exhibit slightly lower latency (0.48 ms), they suffer from a significant performance gap (F1-score: 0.850 vs. our 0.950).

The high efficiency of DB-PG stems from its optimized architecture: (1) the dual-branch structure decomposes the problem into manageable sub-tasks, avoiding the quadratic complexity of full attention mechanisms; and (2) the dynamic graph generation relies on efficient Gaussian kernels rather than heavy matrix factorization. With sub-millisecond latency and ultra-low memory requirements, DB-PG is ideally suited for real-time monitoring and edge computing scenarios in Industrial IoT (IIoT) environments, where resources are constrained and immediate response is critical.

## 5. Conclusions

This paper presents DB-PG, a dual-branch graph learning framework for anomaly and cyberattack detection in industrial sensor time series. The model jointly captures spatial sensor correlations via adaptive graph learning and long-range temporal dependencies through a hierarchical patch-based attention–GRU structure. To regularize the graph structure and improve prediction quality, we employ a composite loss that integrates MSE prediction loss, graph sparsity and smoothness constraints, as well as SSIM loss. The anomaly score further combines prediction errors and graph structure variations across consecutive patches to enhance detection robustness. The experimental results show that DB-PG outperforms state-of-the-art methods on real-world industrial datasets, validating its effectiveness and practicality for real-world monitoring systems. In future work, we plan to extend the model toward online inference and improve the interpretability of dynamic graph structures.

## 6. Future Work

While this study focuses on multivariate time series from physical sensors, industrial cyberattacks often leave traces across multiple heterogeneous data sources. Therefore, a promising future direction is to extend our framework towards multimodal industrial intrusion detection. Specifically, we plan to integrate network traffic logs (e.g., ModbusTCP packets) and device syslogs alongside time-series sensor readings. The primary challenge lies in fusing these disparate modalities, where time series capture physical dynamics, traffic reflects communication patterns, and logs provide semantic operational context. We envision a heterogeneous graph neural network architecture to model the complex inter-modal relationships, enabling the detection of sophisticated attacks (e.g., multi-stage advanced persistent threats) that evade single-modality detection by manifesting simultaneously at the physical and cyber layers.

## Figures and Tables

**Table 1:** Statistics of the datasets.

Datasets	SWaT	WADI	HAI	BATADAL
Features	51	112	86	43
Attacks	41	15	58	14
Training size	496,190	1,208,990	1,004,400	8176
Testing size	449,310	172,190	361,080	6266
Anomaly rate	12.14%	5.75%	57.82%	14.35%

**Table 2:** Experimental configuration details.

Notation	Description	Value
$N_{B}$	Batch size	32
$N_{E}$	Number of epochs	5
η	Learning rate	$10^{-4}$
*w*	Look-back window	96
*l*	Prediction horizon	96
$L_{P}$	Number of patches	4
$S_{P}$	Patch stride	8
$S_{w}$	Moving average window	13
$f_{k}$	Number of top frequency	5
*d*	Hidden dimension	32
$g_{B}$	Number of GNN layers	2
*B*	Number of DB-PG blocks	3
$\lambda_{1},\lambda_{2},\lambda_{3},\lambda_{4}$	Weight of the loss	0.4, 0.3, 0.1, 0.2
$\alpha_{1},\alpha_{2},\alpha_{3}$	Weight of the score	0.6, 0.3, 0.1

**Table 3:** Anomaly detection accuracy in terms of precision (%), recall (%), F1-score (%) and F1-score (without point adjustment) on datasets with ground-truth-labeled anomalies on SWaT and WADI. The best and second-best performances are highlighted in bold and underline, respectively.

Parameter	SWaT				WADI			
	P	R	F1	$\hat{F1}$	P	R	F1	$\hat{F1}$
MAD-GAN	97.89	78.98	87.42	77.70	92.10	69.40	79.15	55.56
OmniAnomaly	99.96	85.49	92.16	**82.60**	81.15	67.01	74.14	41.71
ModernTCN	91.83	92.98	91.86	76.97	78.62	57.69	66.55	50.63
MTAD-GAT	**99.99**	65.66	79.27	60.22	88.11	59.31	70.89	43.75
GDN	99.35	85.12	91.68	81.14	89.30	54.70	67.84	57.53
TranAD	**100.00**	65.14	79.26	66.69	92.62	57.40	70.87	41.24
ATransformer	98.26	85.90	85.90	71.42	77.93	63.61	73.17	35.44
FED	98.79	88.35	93.28	80.69	95.61	74.23	84.78	60.20
USAD	99.99	66.70	80.02	79.20	92.48	63.97	75.63	43.15
TSMixer	98.84	91.97	**95.28**	75.73	**97.99**	81.22	88.40	37.46
DCdetector	98.83	85.34	91.59	76.61	95.94	78.53	86.36	53.17
MODEM	89.42	**93.08**	91.21	77.76	82.62	67.40	72.23	38.27
ImDiffusion	89.88	84.65	87.09	79.28	90.58	**84.34**	**88.87**	70.57
CATCH	83.85	81.29	82.55	80.53	87.88	77.04	82.04	**77.76**
**Ours**	98.64	**94.60**	**96.58 ± 0.12**	**84.91 ± 0.57**	**98.08**	**84.01**	**90.50 ± 0.21**	**81.52 ± 0.49**

**Table 4:** Anomaly detection accuracy in terms of precision (%), recall (%), F1-score (%) and F1-score (without point adjustment) on datasets with ground-truth-labeled anomalies on HAI and BATADAL. The best and second-best performances are highlighted in bold and underline, respectively.

Algorithm	HAI				BATADAL			
	P	R	F1	$\hat{F1}$	P	R	F1	$\hat{F1}$
MAD-GAN	96.45	81.20	88.17	76.54	90.15	71.30	79.63	68.42
OmniAnomaly	99.10	86.75	92.51	81.33	83.40	68.90	75.44	74.18
ModernTCN	93.20	90.15	91.66	77.82	76.80	60.45	67.63	59.71
MTAD-GAT	**99.98**	67.80	80.75	61.45	85.60	62.10	72.03	68.60
GDN	98.80	84.30	90.98	80.12	87.20	58.40	69.92	54.33
TranAD	92.47	66.40	77.29	65.88	94.10	60.20	73.39	62.55
Anomaly Transformer	97.50	86.10	91.46	70.25	79.80	65.20	71.76	56.90
FED	97.20	89.60	93.23	79.54	**96.80**	75.40	84.73	67.05
USAD	**99.95**	67.20	80.25	78.33	91.30	66.80	77.10	54.82
TSMixer	97.10	**93.80**	**95.42**	76.20	**96.80**	79.10	**87.09**	69.25
DCdetector	99.05	83.60	90.69	75.44	93.40	76.80	84.33	56.64
MODEM	90.10	92.40	91.24	78.11	80.50	70.10	74.96	50.15
ImDiffusion	88.20	85.70	86.94	78.02	88.90	**82.60**	85.62	68.40
CATCH	85.60	79.30	82.33	**81.95**	85.20	74.10	79.27	**76.33**
**Ours**	97.80	**94.20**	**95.97**	**83.10**	**97.50**	**82.90**	**89.61**	**79.60**

**Table 5:** Anomaly detection accuracy in terms of precision (%), recall (%), and F1-score of FDG and its variants on SWaT and WADI. The best and second-best performances are highlighted in bold and underline, respectively.

Method	SWaT			WADI		
	P	R	F1	P	R	F1
**Ours (Full)**	**98.64**	**94.60**	**96.58**	**98.08**	**84.01**	**90.50**
w/o dual-branch (period-only)	90.61	72.82	80.75	82.57	62.7	73.99
w/o dual-branch (trend-only)	91.81	74.51	82.26	80.48	64.36	71.52
w/o FreqDecomp (single branch with patch)	92.55	85.74	89.01	84.33	66.52	74.66
w/o adaptive graph (fully connected graph)	94.65	84.22	89.10	83.41	67.85	74.82
w/o frequency gating	95.46	87.54	92.85	85.74	75.73	80.42
w/o Intra-Attn	97.66	93.20	95.38	95.89	85.36	90.32
w/o Inter-Attn	96.92	88.38	92.45	81.27	78.63	79.93
w/o graph variation loss	96.27	94.11	95.17	94.31	75.53	83.88
GCN	95.19	94.47	94.82	93.79	77.64	84.95

**Table 6:** Anomaly detection accuracy in terms of precision (%), recall (%), and F1-score of FDG and its variants on HAI and BATADAL. The best and second-best performances are highlighted in bold and underline, respectively.

Method	HAI			BATADAL		
	P	R	F1	P	R	F1
**Ours (Full)**	**97.80**	**94.20**	**95.97**	**97.50**	**82.90**	**89.61**
w/o dual-branch (period-only)	89.75	73.20	80.68	81.42	63.85	71.54
w/o dual-branch (trend-only)	90.24	76.38	82.75	79.63	65.12	71.64
w/o FreqDecomp (single branch with patch)	90.18	84.92	87.47	85.47	64.30	73.38
w/o adaptive graph (fully connected graph)	95.32	83.76	89.14	82.55	68.40	74.78
w/o frequency gating	94.88	86.42	90.44	86.15	74.68	79.96
w/o Intra-Attn	96.54	92.18	94.31	94.20	84.76	89.24
w/o Inter-Attn	95.60	87.24	91.24	80.35	77.42	78.86
w/o graph variation loss	92.35	90.07	91.19	95.91	79.27	86.80
GCN	96.43	93.71	95.05	95.66	78.83	86.43

**Table 7:** Comparison of alternative attack detection techniques per event. The best and second-best performances are highlighted in bold and underline, respectively.

Method	SWaT	WADI	HAI	BATADAL
MAD-GAN	16	8	27	10
OmniAnomaly	20	6	30	8
ModerTCNn	20	6	17	6
MTAD-GAT	22	7	40	10
GDN	28	8	**46**	**11**
TranAD	22	6	36	8
Anomaly Transformer	24	6	30	7
FED	25	9	35	10
USAD	13	7	25	10
TSMixer	**29**	**10**	**46**	9
DCdetector	23	**10**	45	**11**
MODEM	23	8	38	8
ImDiffusion	24	8	39	7
CATCH	22	7	40	7
**Ours**	**32**	**11**	**47**	**11**

**Table 8:** Comparison of alternative attack detection techniques per event (SWaT). Numbers underlined denote detectable anomalies.

Stage	Attacks	P	R	F1
P1	1,2,3,21,26,30,33,34,35,36	100	94	97
P2	6,24,29,30	100	56	72
P3	7,8,13,14,17,23,26,27,28,32,41	100	99	99
P4	10,11,22,25,27,31,38,39,40	99	83	91
P5	4,19,20,22,37,38,39	100	65	78
P6	23	100	94	97

**Table 9:** Comparison of alternative attack detection techniques per event (WADI). Numbers underlined denote detectable anomalies.

Attacks	P	R	F1
1,2,3,4,5,6,7	99	96	97
8,9,10,11,12,13,14	100	94	97

**Table 10:** Comparison of alternative attack detection techniques per event (HAI). Numbers underlined denote detectable anomalies.

Attacks	P	R	F1
1,2,3,4,5,6,7,8,9,10,11	98	96	97
8,9,10,11,12,13,14,15,16,17,18,19,20,21,22	96	92	94
23,24,25,26,27,28,29,30,31,32,33,34,35,36,37	96	92	94
38,39,40,41,42,43,44,45,46,47,48,49,50,51,52	95	93	93
53,54,55,56,57,58	99	97	97

**Table 11:** Comparison of alternative attack detection techniques per event (BATADAL). Numbers underlined denote detectable anomalies.

Attacks	P	R	F1
1,2,3,4,5,6,7	96	91	93
8,9,10,11,12,13,14	95	94	94

**Table 12:** Hyperparameter sensitivity of the proposed method on the SWaT dataset.

Seasonal Window	$f_{k}=1$	$f_{k}=2$	$f_{k}=3$	$f_{k}=4$	$f_{k}=5$	$f_{k}=6$	$f_{k}=7$	$f_{k}=8$
*S_w_* = 7	86.06	87.31	88.99	89.19	90.05	88.97	87.46	87.55
*S_w_* = 9	89.47	88.71	90.87	90.45	91.28	91.52	89.88	89.25
*S_w_* = 11	90.48	89.79	90.47	93.51	93.83	91.71	92.70	89.99
*S_w_* = 13	92.80	91.20	93.55	94.18	96.58	93.30	92.37	91.98
*S_w_* = 15	91.17	89.90	92.39	92.63	93.44	92.74	91.94	90.25
*S_w_* = 17	88.68	89.42	89.74	91.92	92.23	89.58	89.28	89.47

## Data Availability

The data presented in this study are available on the source mentioned in the text.
